# Effect of eccentric fixation on the steady-state pattern electroretinogram

**DOI:** 10.1007/s10633-024-09967-w

**Published:** 2024-02-28

**Authors:** Evelyn B. N. Friedel, Julia Haldina, Kathrin Nickel, Michael Bach, Ludger Tebartz van Elst, Sven P. Heinrich

**Affiliations:** 1 Eye Center, Medical Center – University of Freiburg, Faculty of Medicine, University of Freiburg, Freiburg, Germany; 2 Department of Psychiatry and Psychotherapy, Medical Center – University of Freiburg, Faculty of Medicine, University of Freiburg, Freiburg, Germany; 3 Faculty of Biology, University of Freiburg, Freiburg, Germany

**Keywords:** Pattern electroretinogram, PERG, Steady-state PERG, Eccentricity, Fixation, Misfixation

## Abstract

**Purpose:**

The steady-state pattern electroretinogram (ssPERG) is used to assess retinal ganglion cell function in a variety of research contexts and diagnostic applications. In certain groups of patients or study participants, stable central fixation of the stimulus is not guaranteed. The present study aimed at assessing the effects of misfixation on the ssPERG response to checkerboard reversal stimuli.

**Methods:**

Using two check sizes (0.8° and 15°), we compared ssPERG responses for several amounts of fixation deviation, ranging from 0° to 19° horizontally and from 0° to 14° diagonally. The stimulus area extended to 15° eccentricity, stimulus reversal rate was 15/s.

**Results:**

Up to around 7° eccentricity, there was no sizable effect of fixation deviation under most conditions. Effects were somewhat larger for nasal than for temporal deviation, in particular for small checks. Diagonal deviation was associated with a response to luminance onset/offset at 7.5 Hz (subharmonic of the reversal rate), most prominently when the interior of a large check was fixated.

**Conclusion:**

Generally, moderate inaccuracies of fixation do not have a sizable effect on ssPERG amplitude. However, with large checks, the luminance response has to be considered.

## Introduction

The pattern electroretinogram (PERG) is used to record ganglion cell responses in a variety of clinical and research contexts [1, 2]. This includes not only testing of macular function [3] and early detection of glaucoma [4, 5], but also assessment of drug effects [6–9]. In cases of suspected malingering, the combination of a normal PERG and an altered visual evoked potential (VEP) response may provide decisive evidence for an organic disorder [10]. Recently, the PERG has received increased interest as a potential diagnostic biomarker for psychiatric disorders [11–15].

In contrast to the flash ERG, the PERG uses pattern reversal stimuli, which are particularly suitable to target retinal ganglion cells via local contrast inversion without change in mean luminance [16]. With every reversal of the pattern stimulus, local luminance responses on the retina cancel out, and only nonlinear components remain, which constitute the PERG [16]. The steady-state variant (> 10 reversals per second (rps)) of the PERG (ssPERG) is frequently used if the response magnitude, rather than the shape of the response curve, is of primary interest. It allows for efficient recording and relatively simple frequency-space response detection and statistical assessment [17].

In several fields of application, accurate central fixation is not always guaranteed. For instance, patients with central visual field defects may rely on eccentric fixation [18]. Studies in patients with psychiatric disorders (e.g., schizophrenia or depression) showed differences in the ability to maintain proper fixation compared to controls [19–22], and in cases of malingering, patients may choose to fixate improperly.

Effects of improper fixation have previously been assessed for different types of electrophysiological exams, such as multifocal ERGs [23, 24] and acuity VEPs [25]. For the transient PERG, Persson and Wagner [26] did not find a sizable effect with fixation at 4° eccentricity and check sizes of 24 arcmin.

The present study was designed to quantify the effects of different amounts of deviation from central fixation on the ssPERG in order to provide a basis for judging the relevance of fixation inaccuracies in clinical practice and research applications. We performed two experiments.

In Experiment 1, we assessed purely *horizontal* misfixation. In this case, as the gaze direction changed along the edge between checks, luminance changes at the time of the checkerboard reversals continued to be balanced, as a switch from black to white above the eccentric fixation point was compensated by a switch from white to black below the fixation point. However, the location of vertical edges in the stimulus changed on the retina and the stimulus pattern as a whole was displaced.

In Experiment 2, fixation was varied along the *diagonal*. This resulted in the gaze being directed at points that were located in the interior of a check. Thus, when considering the vicinity of the fovea, the reversals of the stimulus resulted in locally unbalanced luminance changes. Due to the eccentricity dependence of retinal circuitry [27], we expected this effect not to be fully balanced across retinal locations, potentially resulting in an undesired luminance response in the ssPERG. The imbalance should be more pronounced for larger checks.

## Methods

### General methods

#### ssPERG recording and stimulation procedure

Stimulation and recording was conducted with the EP2000-System [28] following the recommendations of the International Society for Clinical Electrophysiology and Vision (ISCEV) for PERG recordings [1]. ssPERG was recorded from both eyes simultaneously using DTL (Dawson, Trick and Litzkow)-like electrodes [29] placed at the lower limbus of each eye. Reference electrodes were attached to both ipsilateral canthi, with an ear-clip as ground electrode. Signals were amplified (50-fold), filtered with a first order band pass (5–100 Hz) and digitized at 1 kHz with 16 bit resolution.

Participants were placed at a distance of 57 cm from a 17-inch CRT (Cathode Ray Tube)-monitor (800 × 600 pixels, 75 Hz refresh rate). The stimuli (600 × 600 pixels) covered an area of 30° × 30° (i.e., extending to 15° horizontal and vertical eccentricity with central fixation), which corresponds to the “large field PERG” (larger than the regular 15° × 15° (± 3°) field size) as described in the ISCEV recommendations for standard PERG recordings [1].

Black/white checkerboards served as pattern stimuli with a mean luminance of 45 cd/m^2^ and a Michelson contrast of near 100% with a reversal rate of 15/s, which is suggested in the PERG-standard [1] for glaucoma studies and has been used previously for early detection of glaucoma [30].

In both experiments, two different check sizes (0.8° and 15°) were used in alternating blocks, with each block lasting about 5.3 s and consisting of 5 consecutive sweeps of 1065 ms duration. Each recording run consisted of 8 cycles of the alternating block presentation, resulting in a total of 40 artifact free sweeps per check size for averaging. Due to the large number of eccentric fixation locations in Experiment 1, only one recording run (40 sweeps) per check size and fixation location was performed. In Experiment 2, two recording runs (2 × 40 sweeps) were conducted, and the results averaged for each check size and each fixation target. A threshold of ± 120 µV was applied for automated rejection of artifact-contaminated individual sweeps.

#### Analysis

Offline analysis was performed with Igor Pro 7 (Wavemetrics Inc.). A discrete Fourier transformation was applied to the sweep average after removal of any linear trend (e.g., originating from baseline drifts) [17]. For display, a 40-Hz low-pass filter was applied to the time-series data. ssPERG amplitudes were extracted from the frequency spectrum at 15 Hz (corresponding to the reversal rate of 15/s of the stimulus) and, in Experiment 2, also at half the reversal rate in seconds to be able to estimate possible luminance responses (at the subharmonic frequency of 7.5 Hz). Amplitudes were subsequently corrected for noise [17] and tested for significance as described by Meigen and Bach [31].

In Experiment 1, data from each eye were considered separately to determine whether there was a difference in effect between inward vergence (nasal fixation deviation) and outward vergence (temporal fixation deviation). In Experiment 2, ssPERG amplitudes were averaged across eyes and recording runs.

To estimate the degree of ssPERG amplitude reduction with increasing distance from the central fixation point, without confounding effects from inter-individual variability, we normalized ssPERG amplitudes for all participants in relation to central fixation by computing the ratio (ssPERG eccentric fixation point / ssPERG central fixation point). Medians were determined and the corresponding 95% confidence intervals (CI) were estimated via bootstrapping (10,000 replicates). In Experiment 1, we expected an amplitude reduction with horizontal fixation deviation but no luminance responses; therefore, we chose one-sided CIs for the analysis. In Experiment 2, two-sided CIs were calculated.

### Specific methods of Experiment 1

#### Participants

Sixteen (four male) neurotypical, healthy participants without known ophthalmological diseases, except for refraction errors, took part in the experiment. The age range was 21–44 years, with a mean age of 29 years (standard deviation: 7 years). For inclusion, a minimum monocular decimal visual acuity of 0.8 had to be achieved in the Freiburg visual Acuity and Contrast Test (FrACT) [32], using refractive correction if necessary.

#### Fixation deviation

**Fig. 1:**
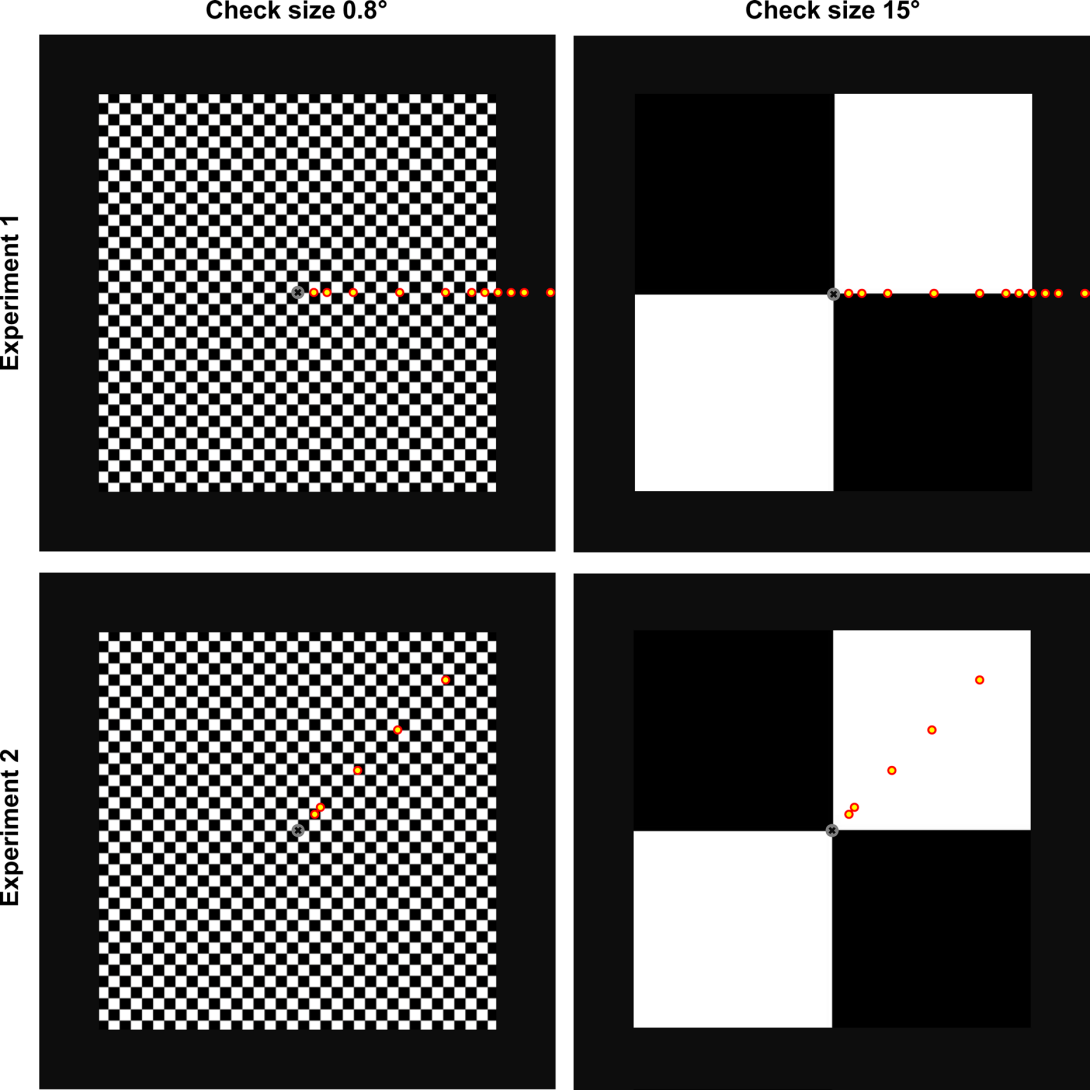
Schematic representation of both checkerboard patterns (0.8° and 15°) and the locations of the different fixation targets (yellow-filled red circles). Experiment 1 (horizontal fixation deviation; left or right): 0°, 1°, 2°, 4°, 7.5°, 11°, 13°, 14°, 15°, 16°, 17°, and 19°. Experiment 2 (diagonal fixation deviation toward the upper right corner): 0°, 1.7°, 2.3°, 7°, 11° and 14°. During central fixation, participants had to announce the random small digits that occasionally appeared in the original fixation mark (central circular disk with a cross inside), eccentric targets were only marked by small dots

In order to quantify effects from inappropriate fixation during ssPERG recordings, we marked eleven eccentric fixation points on the horizontal axis at 0°, 1°, 2°, 4°, 7.5°, 11°, 13°, 14°, 15°, 16°, 17°, and 19° eccentricity. As exemplarily shown in Fig. 1, the set of fixation marks thus included the border of the stimulus area (15° eccentricity) as well as points outside the stimulus area (16°, 17°, and 19° eccentricity). For each eccentricity, one recording run (40 sweeps) of ssPERG was acquired. Participants were instructed to direct their gaze to the respective fixation mark without turning their head. The sequence of eccentricities was pseudo-randomized across participants, and the direction of eccentricity (left or right) was balanced across participants.

Depending on the combination of the direction of fixation deviation (left or right) and the recorded eye (also left or right), the fixation conditions were relabeled as “nasal” or “temporal”, with “nasal” corresponding to inward vergence and “temporal” corresponding to outward vergence. Importantly, by limiting eccentric fixation to horizontal deviations, all fixation targets were located on an edge between checks (except for those targets that were outside the stimulus area). Thus, even with eccentric fixation, there is no relevant overall luminance change when the checkerboard reverses.

### Specific methods for Experiment 2

#### Participants

Twelve (three male) neurotypical, healthy participants without known ophthalmological diseases, except for refractive errors, took part in the experiment. The age range was 22–42 years, with a mean age of 28 years (standard deviation: 6 years). All participants had a monocular decimal visual acuity of ≥ 0.8 (with refraction if necessary) as confirmed with the FrACT.

#### Fixation deviation

As shown in Fig. 1, fixation targets were presented along the 45°-diagonal toward the upper right. The distances from the center were tested: 0°, 1.7° (center of a 0.8° check), 2.3° (where the corners of four 0.8° checks touch), 7° ($${\raise0.7ex\hbox{$1$} \!\mathord{\left/ {\vphantom {1 3}}\right.\kern-0pt} \!\lower0.7ex\hbox{$3$}}$$ of a 15° check), 11° (center of a 15° check), and 14° ($${\raise0.7ex\hbox{$2$} \!\mathord{\left/ {\vphantom {2 3}}\right.\kern-0pt} \!\lower0.7ex\hbox{$3$}}$$ of a 15° check).

## Results

### Experiment 1

**Fig. 2:**
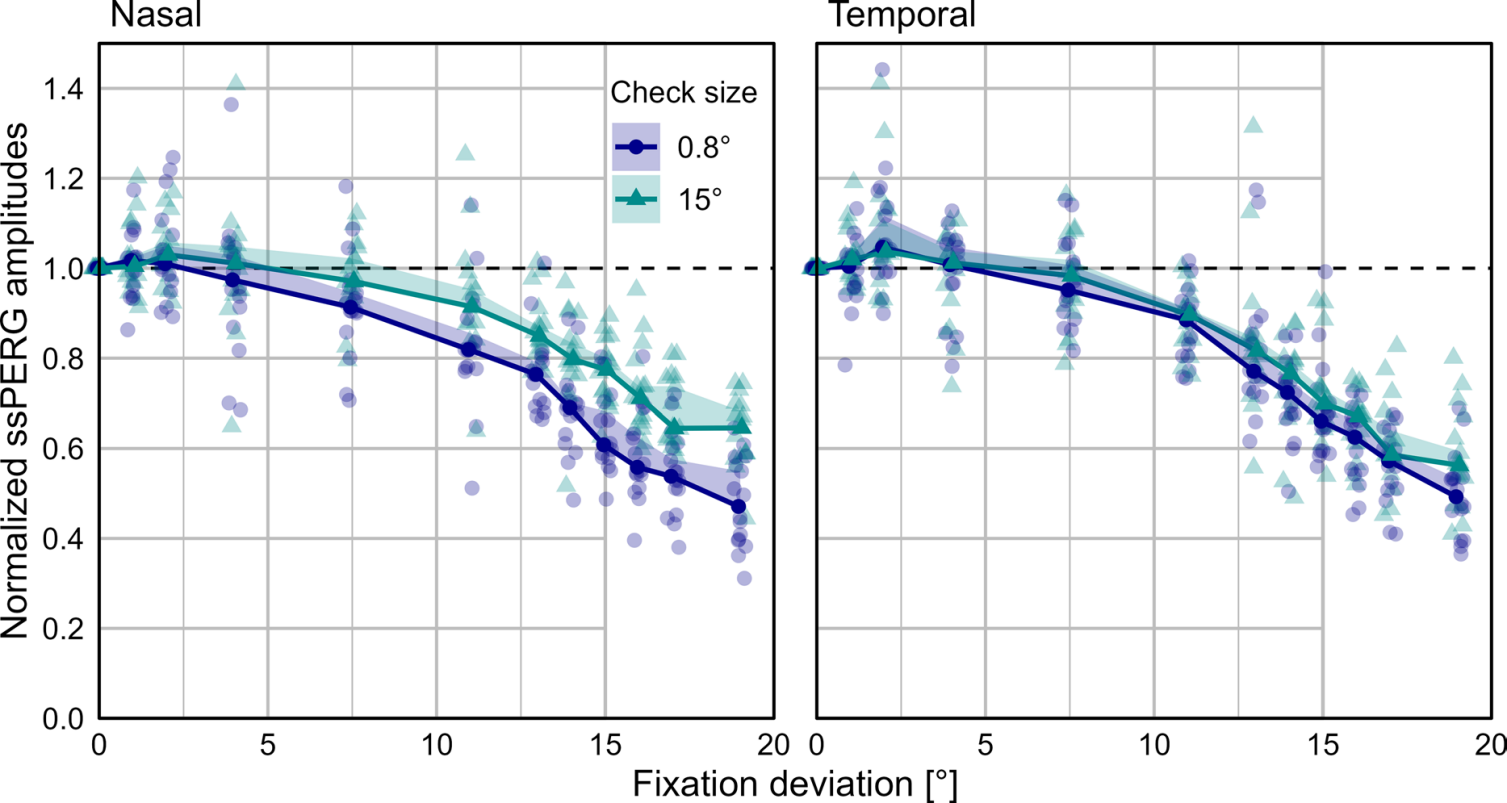
Medians and (median-based) one-sided bootstrapped 95% confidence intervals for the normalized ssPERG amplitudes, which were calculated as ratios relative to the central fixation (0°) for all eccentric fixation points (1°–19°). Data for both check sizes (0.8° and 15°) and both deviation sides (left graph nasal and right graph temporal) are depicted. Fixation points  > 15° were located outside the stimulus area

ssPERG amplitudes in eccentric fixation conditions remained nearly unaffected (less than 10% decline) up to about 4° departure from central fixation (Fig. 2), independent of the check size presented. At a fixation deviation of 7.5°, the median ssPERG amplitude for the larger checks (15°) was only sightly unattenuated (< 10% reduction), whereas the ssPERG from the smaller check (0.8°) is somewhat more affected (≈ 10% reduction), in particular with nasally deviation. Toward higher eccentricities (> 7.5°), the median of the normalized ssPERG amplitudes declined continuously. Generally, ssPERG amplitudes from the temporal conditions seemed to be affected similarly for both pattern stimuli (0.8° and 15°), whereas responses from the nasal conditions tended to be more affected in case of the small check size (0.8°), compared to the larger check size (15°).

### Experiment 2

**Fig. 3:**
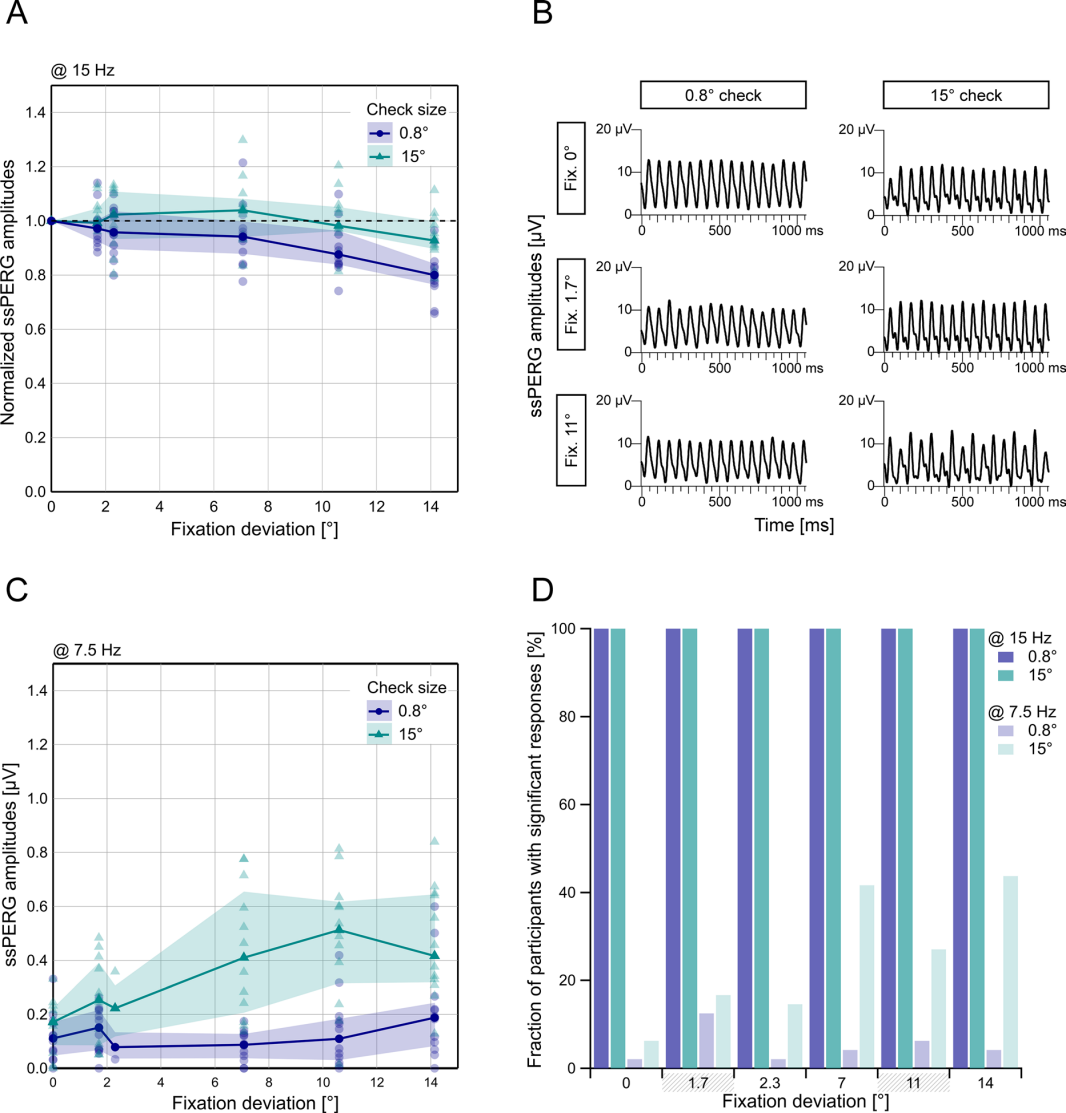
Results from Experiment 2 for diagonal fixation deviation toward the upper right corner (fixation marks: 0°, 1.7°, 2.3°, 7°, 11° and 14°) for both check sizes (0.8° and 15°). **a** Median and bootstrapped two-sided 95% confidence intervals for normalized ssPERG amplitudes averaged across eyes. **b** Exemplary ssPERG sweeps for central (0°), 1.7° and 11° fixation points from one individual, showing increased subharmonic responses (additional peaks) at 7.5 Hz with 15° checks, compared to 0.8° checks. **c** Median and two-sided 95% confidence intervals for ssPERG amplitudes from all participants in µV extracted at 7.5 Hz from the frequency spectrum (subharmonic responses) and averaged across eyes. **d** Fraction of participants [%] with significant ssPERG responses for both eyes, both recording runs and analyzed frequencies (15 Hz as stimulus reversal rate and 7.5 Hz for possible subharmonic luminance responses)

Up to around 7° diagonal fixation deviation, ssPERG amplitudes at 15 Hz remained relatively stable (< 10% change in amplitude). For the large checks (15°), this continued toward larger deviations (e.g., < 10% decline with 11° fixation deviation) (Fig. 3A), while ssPERGs to the smaller checks (0.8°), seemed to be slightly more affected by increasing fixation deviation (> 7°, e.g., > 10% attenuation with 11° fixation deviation).

Although the ssPERG elicited by the larger checks (15°) shows a more “stable” response with increasing fixation deviation showing up at 15 Hz, an inspection of the averaged ssPERG sweeps (Fig. 3b) reveals that larger and smaller response peaks are alternating when the interior of a large check (15° check; 11° deviation) was fixated. This results in a marked subharmonic response component at 7.5 Hz, which was more pronounced for the larger checks (15°) and less prominent for the smaller checks (0.8°) (Figure 3c shows median amplitudes from all participants). The additional subharmonic responses at a frequency of 7.5 Hz were statistically significant in a number of participants (Fig. 3d). For both check sizes, subharmonic response amplitudes were largest when fixation marks fell in the center of a check (Fig. 3c; 0.8° check and 1.7° deviation; 15° check and 11° deviation; compare Fig. 1).

## Discussion

The present data show that moderate fixation inaccuracies, in the order of 7 degrees, have a nearly negligible effect on the ssPERG amplitude. In particular, when the check size is large and the gaze does not fall directly in the center of a check, ssPERG amplitudes remain robust, for both tested directions of misfixation. The exact dependence of ssPERG amplitude and fixation eccentricity will obviously depend on the overall extent of the stimulus area, as the position of the outer boundary of the stimulus area can be assumed to be the main determinant of ssPERG amplitude loss. This is consistent with previous findings showing that stimulation at higher eccentricities has a relatively lower contribution to the generation of the PERG [33]. The amount of fixation inaccuracy that is tolerable in a given research context or in diagnostic use will depend on the expected effect sizes. The observed minor ssPERG amplitude alterations (< 10%) with moderate misfixation are comparable with normal PERG amplitude variations due to inter-session (coefficient of variation 6⎼16%) or diurnal (coefficient of variation ≈ 10%) variability [34].

Comparing both directions of horizontal deviation, only minor differential effects were observed on the ssPERG responses from the different eyes and thus from nasal and temporal sides. Two previous studies however reported larger PERG amplitudes with nasal compared to temporal hemifield stimulation [35, 36] and suggested that this might be due to the higher number of nasally distributed retinal ganglion cells in the peripheral retina [37]. In our case, naso-temporal differences were rather small but seemed to be stimulus-specific. With temporal fixation deviation (rightward deviation of the right eye or leftward deviation of the left eye), the ssPERG signal decreases similarly with both check sizes when misfixation increases. With nasal fixation deviation, however, ssPERG responses to the finer checks seemed to be somewhat more affected with increasing fixation deviation, compared to the responses to the larger checks. Considering the complex relationship between PERG responses and the various stimulus parameters, including check size, a definite interpretation of this effect is beyond the present study.

An inequality of the responses to both checkerboard polarities was observed in Experiment 2, particularly if the interior of a large check was fixated. This is most likely a luminance effect that originates at least partly from cell types other than ganglion cells and may need to be considered when interpreting ssPERG findings in terms of ganglion cell function. As the respective frequency of 7.5 Hz was relatively close to the lower cut-off frequency of the bandpass filter of our set-up (5 Hz), the luminance responses were possibly somewhat attenuated.

Using a relatively large stimulus extent, as in the present study (30° × 30°), can be useful when fixation problems are expected (as implicated by Sakaue et al. [38] and Junghardt et al. [39]), if the purpose of the recording does not require a smaller stimulated area. We estimate that the present results would in principle also hold for the standard stimulus extent (15° mean width and height) with the acceptable angle of misfixation scaled correspondingly (e.g., up to around 3° misfixation).

The present study addressed the question of static fixation inaccuracies. Clearly, eye movements might have additional undesired effects on ssPERG signal quality, arising from the electroretinographic response to the moving retinal image [40] and the intrusion of electrooculographic artifacts [41]. However, standard threshold-based artifact detection combined with frequency-domain response analysis should normally ensure that eye movements do not have a sizable effect on the test outcome.

In summary, the present study suggests that moderate fixation inaccuracies do not have a major impact on the outcome of ssPERG recordings.

## Data Availability

All data collected, analyzed, or generated in this study are available upon request from the corresponding author EF.
